# Rationale and Design of BeatNF2 Trial: A Clinical Trial to Assess the Efficacy and Safety of Bevacizumab in Patients with Neurofibromatosis Type 2 Related Vestibular Schwannoma

**DOI:** 10.3390/curroncol28010071

**Published:** 2021-01-31

**Authors:** Masazumi Fujii, Masao Kobayakawa, Kiyoshi Saito, Akihiro Inano, Akio Morita, Mitsuhiro Hasegawa, Akitake Mukasa, Takafumi Mitsuhara, Takeo Goto, Shigeru Yamaguchi, Takashi Tamiya, Hirofumi Nakatomi, Soichi Oya, Fumiaki Takahashi, Taku Sato, Mudathir Bakhit, on behalf of the BeatNF2 Trial Investigators

**Affiliations:** 1 Department of Neurosurgery, Fukushima Medical University, Fukushima 960-1247, Japan; kiyoshis@fmu.ac.jp (K.S.); tak-s@fmu.ac.jp (T.S.); m-bakhit@fmu.ac.jp (M.B.); 2 Medical Research Center, Fukushima Medical University, Fukushima 960-1247, Japan; mkobaya@fmu.ac.jp (M.K.); ainano@fmu.ac.jp (A.I.); 3 Department of Neurological Surgery, Nippon Medical School, Bunkyo-Ku, Tokyo 113-8602, Japan; amor-tky@umin.ac.jp; 4 Department of Neurosurgery, Fujita Health University, Toyoake 470-1192, Japan; mhasegawa-nsu@umin.ac.jp; 5 Department of Neurosurgery, Kumamoto University, Kumamoto 860-8555, Japan; mukasa-nsu@umin.ac.jp; 6 Department of Neurosurgery, Graduate School of Biomedical and Health Sciences, Hiroshima University, Hiroshima 739-8511, Japan; mitsuhara@hiroshima-u.ac.jp; 7 Department of Neurosurgery, Osaka City University, Osaka 558-8585, Japan; gotot@med.osaka-cu.ac.jp; 8 Department of Neurosurgery, Hokkaido University, Sapporo 060-0808, Japan; yama-shu@med.hokudai.ac.jp; 9 Department of Neurosurgery, Kagawa University, Takamatsu 760-0016, Japan; tamiya@med.kagawa-u.ac.jp; 10 Department of Neurosurgery, University of Tokyo, Bunkyo-Ku, Tokyo 113-8654, Japan; hnakatomi-tky@umin.org; 11 Department of Neurosurgery, Saitama Medical Center, Kawagoe 350-8550, Japan; sooya-tky@umin.ac.jp; 12 Center for Liberal Arts and Sciences, Iwate Medical University, Morioka 020-0023, Japan; ftakahas@iwate-med.ac.jp

**Keywords:** neurofibromatosis type 2, schwannoma, bevacizumab, clinical trial

## Abstract

**Simple Summary:**

Neurofibromatosis type 2 (NF2) is a rare genetic hereditary disease characterized by multiple central nervous system tumors, most frequently bilateral vestibular schwannomas (VSs). No chemotherapeutic agents are available for clinical use, and surgery and radiotherapy are the only therapeutic options available now. Still, neither treatment option alleviates hearing loss in patients with NF2 and VS; they may even exacerbate it. However, bevacizumab has been reported to be effective in suppressing the tumor’s growth and has shown unprecedented efficacy in improving hearing. We describe a new ongoing and novel clinical trial, BeatNF2, a randomized, double-blinded, placebo-controlled, multicenter trial to assess bevacizumab’s efficacy and safety in patients with NF2. The study’s primary endpoint is improved hearing function 24 weeks after the beginning of the treatment protocol.

**Abstract:**

Neurofibromatosis type 2 (NF2) causes bilateral vestibular schwannomas (VSs), leading to deafness. VS is treated by surgery or radiation, but neither treatments prevent hearing loss. Bevacizumab was found to be effective in suppressing the tumor’s growth and may help to improve hearing. We are conducting a randomized, double-blind, multicenter clinical trial to verify the efficacy and safety of bevacizumab in NF2-related VS. The primary objective is to evaluate the efficacy of bevacizumab in improving hearing in the affected ear. One of the secondary objectives is to evaluate bevacizumab’s efficacy in rechallenge treatment in relapsed cases. Sixty patients will randomly receive either bevacizumab or a placebo and will be clinically observed for 48 weeks in the initial intervention phase. In the first half (24 weeks), they will receive either 5 mg/kg of bevacizumab or a placebo drug. In the second half, all patients will receive 5 mg/kg of bevacizumab. If hearing function deteriorated in a patient who had shown improvement during the first phase, a rechallenge dose with bevacizumab would be offered.

## 1. Introduction

### 1.1. General Information

A Randomized Double-Blind Multicenter Trial to Assess the Efficacy and Safety of Bevacizumab for Neurofibromatosis Type 2 (BeatNF2) (https://www.clinicaltrials.jp/cti-user/trial/ShowDirect.jsp?clinicalTrialId=29292), Japanese clinical trial identifier number JapicCTI-194999. https://rctportal.niph.go.jp/en/detail?trial_id=JapicCTI-194999.

Overall dates: from October 2019 to March 2022 (Total of 130 weeks).

Funding organization: Japanese Agency for Medical Research and Development (eRad ID: 19188320, AMED 19lk0201098h0001).

E-mail: beatnf2@fmu.ac.jp.

### 1.2. Principal Investigators

Prof. Kiyoshi Saito, MD, Ph.D. (kiyoshis@fmu.ac.jp) and Dr. Masazumi Fujii, MD, Ph.D. (fujiim@fmu.ac.jp), Department of Neurosurgery, Fukushima Medical University, 1 Hikarigaoka, Fukushima-Shi, 960-1295, Japan. Phone: +81-24-547-1268.

### 1.3. Study Sites

1. Fukushima Medical University Hospital
2. Fujita Medical University Hospital
3. Osaka City University Hospital
4. Hokkaido University Hospital
5. The University of Tokyo Hospital
6. Nippon Medical University Hospital
7. Hiroshima University Hospital
8. Kagawa University Hospital
9. Kumamoto University Hospital
10. Saitama Medical Center

### 1.4. Background

Neurofibromatosis type 2 (NF2) is an autosomal dominant disease that frequently causes bilateral vestibular schwannomas (VSs; also known as acoustic neuromas). Patients with NF2 can suffer from multiple central or peripheral nervous system tumors, including schwannomas, meningiomas, and ependymomas. Although the tumors are pathologically classified as benign, the prognosis for survival is poor because of their multiplicity. Bilateral skull base lesions can cause hearing disturbance and deafness and compress the brainstem, leading to gait disturbances and ataxia [1]. NF2 is designated an incurable disease by the Ministry of Health, Labor, and Welfare in Japan. Between 2009 and 2013, the national NF2 registry of the Japanese Ministry of Health and Welfare documented 807 NF2 patients (44% male, 56% female). In a recent retrospective study, the overall neurological disability in these cases was assessed with a 25-point scoring system encompassing a wide variety of neurologic deficits [2]. In 587 patients for whom longitudinal disability data were available, the significant independent risk factors of progression included age of <25 years at onset, positive family history, positive treatment history, hearing loss, facial paresis, blindness, and hemiparesis. According to an analysis from 1999 of NF2 cases in Japan, the 5-, 10-, and 20-year survival rates were 100%, 87%, and 62%, respectively, among patients in whom age at onset age was >25 years, whereas these survival rates among patients younger than 25 years at onset were 80%, 60%, and 28%, respectively; the prognosis was thus clearly worse for younger patients [3]. Hearing impairment is the most frequent manifestation of the disease; 65% of affected patients have a severe hearing loss of at least 70 dB or more in one ear. The next most common manifestations are spinal cord symptoms, facial palsy, cerebellar ataxia, facial hypoesthesia, dysphagia, and dysarthria [2].

#### 1.4.1. Current Treatment of NF2-Related VS

Either surgical resection or stereotactic radiosurgery are options for NF2 associated VS management; however, neither is sufficient to prevent hearing deterioration. The success of hearing preservation by surgery varies, depending on published reports, but is not satisfactory despite surgeons’ attempts to modify procedures or surgical approaches [4,5,6,7,8,9]. According to an analysis of 165 NF2 surgical operations, the success rate was reported to be as low as 35%, even when surgery was performed by an experienced surgeon [9]. Moreover, hearing function gradually decreases after surgery in the long run, even after the hearing was preserved immediately after surgery [10]. Surgical complications such as facial nerve palsy and ataxia also remain common [1]. Concerning stereotactic radiotherapy, the current evidence suggests that hearing preservation rates for NF2 associated VSs are 33–93% at 5 years and 44% at 10 years [11,12,13,14,15,16,17]; this finding suggests that stereotactic radiotherapy does not produce promising results for long-term preservation of hearing function. Therefore, the tumor is often observed and conservatively managed while hearing is intact but hearing function may deteriorate over time.

#### 1.4.2. Rationale for This Trial

Bevacizumab is a humanized monoclonal antibody that targets vascular endothelial growth factor (VEGF), a critical angiogenic factor involved in physiological and pathological conditions [18]. VEGF expression in NF2-related schwannomas has been reported [19]. In 2009, the VEGF antibody bevacizumab was reported to be effective in suppressing the growth of VS [20], and subsequent follow-up reports have shown unprecedented efficacy, whereby more than 50% of treated patients exhibited tumor shrinkage and, surprisingly, hearing improvement at a rate of just over 50% [21]. This outcome was reinforced in many other studies [22,23]. Bevacizumab is already approved for treating several types of cancer and has an established safety profile [24,25,26,27,28].

On the other hand, although bevacizumab has been shown to suppress tumor growth and preserve hearing effectively, its efficacy may disappear when it is discontinued, and re-exacerbation of the disease may result [29]. Prolonged and continuous bevacizumab treatment for NF2 is associated with side effects such as hypertension and renal dysfunction (proteinuria) [21,30,31,32]. Accordingly, we believe that lengthy, continuous bevacizumab treatment is not preferable for patients with NF2, who, compared with high-grade cancer patients, are expected to survive longer and undergo treatment much longer. In contrast, a rechallenge treatment against re-exacerbation of hearing loss during a follow-up period after the initial treatment would be desirable.

We recently conducted a pilot study to investigate bevacizumab’s therapeutic effect on NF2 associated VS. [29]. Ten patients aged 12 to 45 years (mean: 29.4) who underwent treatment between 2013 and 2018 for 17 tumors were enrolled. All patients received 5 mg/kg of bevacizumab intravenously every two weeks up to four times and were monitored for 3–72 months (mean: 39). A reduction from baseline tumor volume of at least 20% was defined as a therapeutic radiologic response. The radiologic response was detected in 7 tumors (41%). Maximum reduction in tumor volume was identified within three months in 11 tumors and within six months in 3 additional tumors; the reduction at three months was significant compared to the baseline tumor volume. Tumors in patients aged 25 and older showed a significant reduction in mean volume after three months and a significantly lower ratio of posttreatment tumor volume to baseline than younger patients after 3 and 6 months. Eighteen months after initial treatment with bevacizumab, one patient exhibited a 223% increase in tumor volume over baseline; re-administration produced a strong effect, reducing tumor volume to 144% over baseline volume the 21st month. This observation suggests that repeated or interrupted doses of bevacizumab could also impair tumor progression.

Optimal dosages and duration of bevacizumab remain a matter of debate. However, no patient in the pilot study experienced severe side effects such as hypertension or proteinuria, probably because the dosages were limited (5 mg/kg every two weeks, a total of four doses). Other investigators had tried different bevacizumab dosages, including 7.5 mg/kg every three weeks and 5 mg/kg every two weeks, which produced fewer side effects [33]. A recent study showed a 10 mg/kg every two weeks dosage did not add a significant benefit compared with lower dosing regimens [34]. Given these findings, 5 mg/kg of bevacizumab by continued administration for several months, followed by interruption period and restarting before either the tumor volume or hearing function returns to baseline levels, may be justified.

No effective drug treatments for this intractable disease are currently available in general clinical practice. However, no randomized controlled clinical trials have yet been performed to confirm this new therapeutic agent’s efficacy, probably because this disease is quite rare. Accordingly, we planned a randomized placebo-controlled clinical trial to verify the efficacy of bevacizumab in preserving hearing ability in patients with NF2 associated VS, with a follow-up period to assess the drug efficacy duration after discontinuation and the effects of rechallenge doses if applicable. The primary endpoint is the improvement of the maximum word recognition score (WRS) at 24 weeks. Improvement of maximum WRS is defined as an increase of 20% points or greater over baseline score and a WRS of 50% points or above. Maximum WRS was recommended by Plotkin et al. as the main outcome of hearing function for NF2 clinical trials [35] and was adopted for this clinical trial. An improvement of 20% or greater fulfills Plotkin et al.’s hearing response guidelines for all baseline score levels.

## 2. Objectives

Our study’s primary objective is to evaluate bevacizumab’s efficacy in improving the affected ear’s hearing by the targeted lesion. The secondary objectives include evaluating the drug’s efficacy in reducing tumor volumes and assessing rechallenge intervention in tumor relapse cases with deterioration in hearing after a successful initial intervention phase.

## 3. Materials and Methods

### 3.1. Study Overview

The study is a multicenter, randomized, double-blind, placebo-controlled trial conducted in Japan. Patients with NF2 associated VS will be randomly assigned in a 1-to-1 ratio to receive either bevacizumab or placebo. For those receiving bevacizumab, 5 mg/kg will be administered intravenously every two weeks for up to 46 weeks. In the control group, the placebo will be administered intravenously every two weeks for up to 22 weeks, and then 5 mg/kg of bevacizumab will be administered intravenously every two weeks from the 24th to 46th weeks. After 46 weeks, all patients will be monitored; bevacizumab will not be administered unless hearing function deteriorates in the patients who had received bevacizumab for 46 weeks and had shown hearing improvement during that time; rechallenge doses of 5 mg/kg of bevacizumab every two weeks (limited for 12 weeks) will be offered to those patients. The primary endpoint is improved hearing at 24 weeks. The secondary endpoints include several parameters concerning improved hearing and tumor volume reduction at 12, 24, 36, and 48 weeks. Parameters regarding the effects of rechallenge intervention after 12 weeks will also be evaluated.

### 3.2. Sample Study Selection

#### 3.2.1. Inclusion Criteria

The inclusion criteria for this trial are as follows:

1. Either gender, age between 18 and 74, and providing written informed consent. For the participants younger than 20 years old, it must be obtained from a guardian in Japan;
2. A diagnosis of NF2 with either of the following:
  (a) Bilateral VS was confirmed by magnetic resonance imaging (MRI).
  (b) Unilateral VS was confirmed by an MRI study and at least one family member among parents, offspring, or siblings already diagnosed with NF2.
3. Native Japanese language speaking with an ability to be tested with a Japanese version of the WRS;
4. At least a unilateral VS:
  (a) Has not been treated by radiotherapy.
  (b) A hearing test of the affected ear demonstrates both a maximum WRS of 80% or less and pure-tone averages of less than 100 db.
5. Karnofsky performance status score greater than 60% or higher, and general condition allows follow-up in an outpatient clinic;
6. Normal physiological organ functions as confirmed by clinical examination and laboratory tests.

#### 3.2.2. Exclusion Criteria

The exclusion criteria were as follows:

1. History of previous bevacizumab treatment;
2. History of VEGFR vaccine therapy;
3. Surgery is required within the coming six months;
4. Major surgery performed within 28 days before registration or wound not healed after any surgery at the time of registration;
5. A significant traumatic injury or a bone fracture that has not healed;
6. Uncontrolled hypertension at the time of registration (inability to maintain blood pressure below 150 mm Hg (systolic) or 110 mm Hg (diastolic));
7. History of hypertension crisis or hypertensive encephalopathy;
8. Congestive heart failure (New York Heart Association class II or worse) at the time point of registration;
9. Unstable angina or acute myocardial infarction at the time of registration, or a history of either within the previous six months;
10. Symptomatic cerebrovascular diseases (e.g., subarachnoid hemorrhage, cerebral infarction, or transient ischemic attack) at the time of registration or a history of any of these in the previous six months;
11. Vascular disease (e.g., arterial/venous thrombosis or aortic aneurysm) necessitates treatment at the time of registration or any similar history;
12. Usage of antiplatelet drugs, vitamin K antagonists, or anticoagulation drugs of any kind in a therapeutic dose;
13. Hemoptysis of grade 2 or higher, according to the Common Terminology Criteria for Adverse Events (CTCAE) version 4.0, at the time of registration, or a history of this within the previous month;
14. Bleeding tendency (coagulopathy) at the time of registration;
15. Gastrointestinal perforation/fistula or abdominal abscess at the time of registration, or a history of either of these in the previous six months;
16. History of cancer with disease-free periods of less than five years, except for cured basal cell carcinoma, squamous cell carcinoma of the skin, cervical cancer, gastrointestinal tract cancer that has been managed and cured by endoscopic mucosal resection, and WHO grade III primary central nervous system tumors that are stabilized after appropriate treatments;
17. An infectious disease that necessitates treatment with antibiotics, antiviral drugs, or antifungal drugs at the time point of registration;
18. Allergy to drugs made from Chinese hamster ovaries or recombinant humanized antibodies;
19. Contraindications to MRI;
20. A disease is other than NF2 that causes hearing loss in the target ear;
21. In women, pregnancy, breastfeeding, premenopausal status with a positive pregnancy test result, or unwillingness to use contraception during the study period. Women are considered postmenopausal if amenorrhea has continued for more than two years since the last menstrual period;
22. Male patients are unwilling to practice contraception during the study;
23. Determination by the investigators that participation of a patient in this study is inappropriate.

### 3.3. Study Intervention

#### 3.3.1. Initial Intervention up to 22 Weeks

In the bevacizumab group, 5 mg/kg of bevacizumab in 100 mL of normal saline is administered intravenously every two weeks for a total of 12 doses. In the control group, an intravenous infusion of 100 mL of normal saline only is administered.

#### 3.3.2. Initial Intervention from 24 to 46 Weeks

A 5 mg/kg of bevacizumab in 100 mL of normal saline is administered intravenously every two weeks for a total of 12 doses in both groups.

#### 3.3.3. Rechallenge Intervention in Cases of Hearing Deterioration in Bevacizumab Responders

Responders at 48 weeks are defined in the same manner as the primary outcome measure described in Section 3.5.1 below. If a responder experiences deterioration by 20% points or more from the 48 week WRS, a 5 mg/kg of bevacizumab in 100 mL of normal saline is administered intravenously every two weeks total of 6 doses as the rechallenge intervention.

#### 3.3.4. Study Intervention Discontinuation and Patient Withdrawal

If a patient develops certain adverse effects—bleeding of CTCAE grade 2 or worse, proteinuria of CTCAE grade 2 or worse, or hepatic dysfunction of CTCAE grade 3 or worse—the treatment will be temporarily discontinued (i.e., skip a dose). If the patient recovers within two weeks from those adverse effects, the administration will be restarted. Otherwise, if the patient skips two doses because the above-mentioned adverse effect persists, the patient will withdraw from the trial.

If a patient experiences any of the following adverse events, the study intervention will be discontinued permanently and will be able to withdraw from the trial. The adverse effects are grade 4 proteinuria, grade 4 hemorrhage, arterial thrombosis/embolism of any grade, grade 3 or higher venous thrombosis/embolism, grade 3 or higher hypertension uncontrolled by medication, gastrointestinal perforation, cerebral infarction, cerebral hemorrhage (except for intratumoral hemorrhage confirmed by imaging), and reversible posterior leukoencephalopathy syndrome.

### 3.4. Randomization and Blinding

Randomization will be stratified according to age at onset (>25 years old vs. <25 years old) and the number of target lesions (one vs. two) using the minimization method. The investigators will enter subject information into an online registration (allocation) system. Patients will be assigned to receive bevacizumab or placebo according to a predefined randomization algorithm in the computer.

The assignment results will be made known only to prespecified pharmacists in each study site, who will prepare the infusion materials (saline with or without bevacizumab) in anonymous containers. They are prohibited from disclosing the assignment information to anyone else. Except for these pharmacists, all investigators, including the audiologist in charge of the hearing test and the patients, will be unaware of the assignments to avoid bias concerning the therapeutic regimen.

### 3.5. Outcome Measures

#### 3.5.1. Primary Outcome Measure

The primary outcome is the proportion of the patients with improved hearing from baseline at week 24 of the initial intervention. The patient with improved hearing (responder) is defined based on the following criteria:

1. In cases with one target ear, the maximum WRS improves by 20% points or more, and the WRS is 50% points or higher.
2. In bilateral target ears, at least one ear fulfills the above criterion, and the maximum WRSs of the other ear does not deteriorate by 20% points or more.

#### 3.5.2. Secondary Outcome Measures

The following secondary outcomes measures are:

1. The proportion of patients with improved hearing from baseline as assessed by maximum WRS, at weeks 12, 36, and 48 of the initial intervention;
2. The proportion of patients with improved hearing from week 24 as assessed by maximum WRS, at weeks 36 and 48 of the initial intervention, in the control group;
3. The proportion of patients with hearing deterioration from baseline of 20% points or more on the nontargeted side of the lesion, as assessed by maximum WRS, at week 24 of initial intervention;
4. The response rate in tumor volume from baseline as assessed by MRI at weeks 12, 24, 36, and 48 of the initial intervention;
5. The response rate in tumor volume from week 24 as assessed by MRI, at weeks 36 and 48 of the initial intervention, in the control group;
6. The estimated audiogram level (average of four readings) obtained by auditory steady-state response examination at weeks 12, 24, and 48 of the initial intervention;
7. The mean hearing level according to the pure-tone hearing test (average of four readings) at weeks 12, 24, 36, and 48 of the initial intervention;
8. The NF2 severity score at weeks 12, 24, 36, and 48 of the initial intervention;
9. The maximum WRS at week 12 week of the rechallenge intervention in the patients with hearing relapse;
10. The response rate in tumor volume at week 12 of the rechallenge intervention, as assessed by MRI, in the patients with the hearing relapse;
11. The estimated audiogram (average of four readings) obtained by auditory steady-state response examination at week 12 of rechallenge intervention in the patients with hearing relapse;
12. The pure-tone average (average of 4 values) at week 12 of the rechallenge intervention in the patients with hearing relapse;
13. The NF2 severity score at week 12 after starting the rechallenge intervention in patients with tumor relapse;
14. Safety profiles, including drug-related adverse effects, blood and urine examinations, and vital sign measurements.

### 3.6. Trial Organization and Timeline

**Figure 1:**
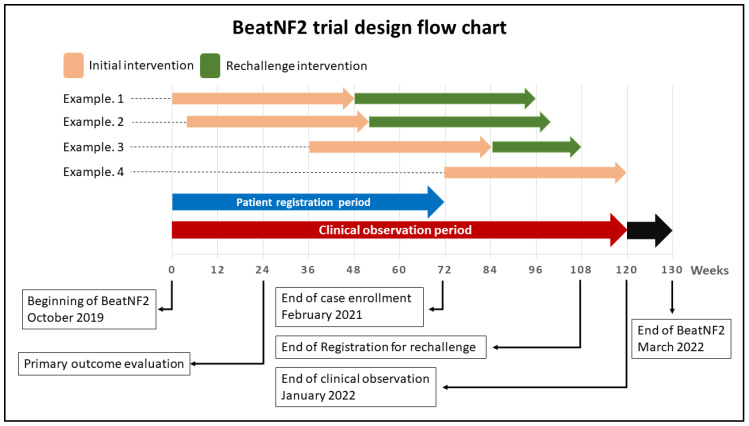
A schematic illustration of the BeatNF2 trial timeline.

**Figure 2:**
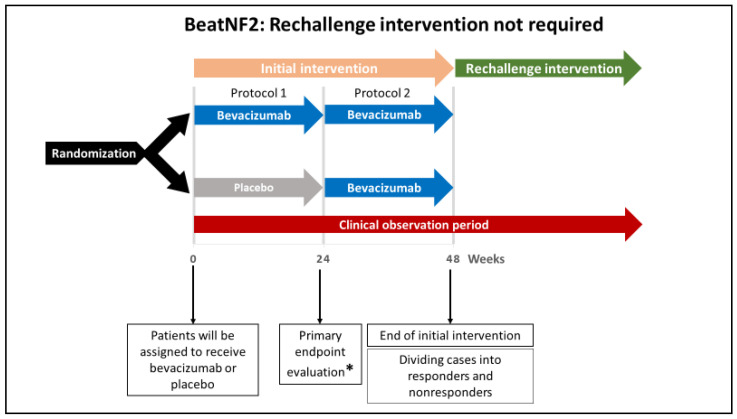
A schematic illustration showing the protocol without rechallenge intervention in the Randomized Double-Blind Multicenter Trial to Assess the Efficacy and Safety of Bevacizumab for Neurofibromatosis Type 2 (BeatNF2) trial. Patients who did not respond to the initial intervention and those who responded without developing deterioration in the hearing will not require rechallenge intervention. * The study’s primary endpoint is improved hearing function 24 weeks after the beginning of the treatment protocol.

**Figure 3:**
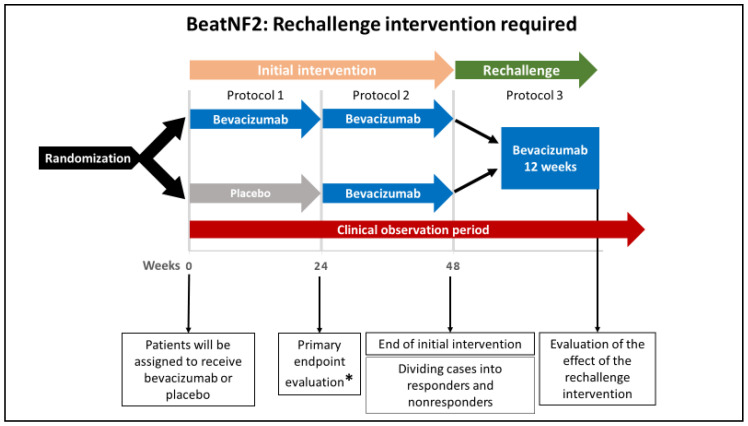
A schematic illustration of rechallenge intervention in the BeatNF2 trial. Patients who respond to the initial intervention but subsequently develop deterioration in hearing receive a rechallenge intervention with six doses of bevacizumab for 12 weeks. This will be followed by evaluating the patient’s hearing function and tumor size at the end of the rechallenge period. * The study’s primary endpoint is improved hearing function 24 weeks after the beginning of the treatment protocol.

The entire BeatNF2 trial design and timeline are shown in Figure 1, Figure 2 and Figure 3.

According to the assignments, patients initially will receive either bevacizumab or placebo every two weeks for 22 weeks (a total of 12 doses; see Figure 2, protocol 1). From week 24 of the initial intervention, patients in both groups will receive bevacizumab every two weeks for additional 22 weeks (see Figure 2, protocol 2). Until this point, the protocol is referred to as the “initial intervention.” If responders are found to develop deterioration in hearing after 48 weeks, a rechallenge intervention with bevacizumab (a total of 6 doses over 12 weeks) will be offered (see Figure 3, protocol 3).

The rechallenge dose, if required, is administered every two weeks (a total of 6 doses). The drug response is evaluated at week 12 of the rechallenge intervention.

Depending on when the patient is recruited, the possibility to offer a rechallenge intervention with its follow-up time is limited. Patients recruited after a specific date cannot be offered the rechallenge intervention of bevacizumab because follow-up duration would be insufficient (see example 4 in Figure 1.)

### 3.7. Study Assessments and Procedures

**Figure 4:**
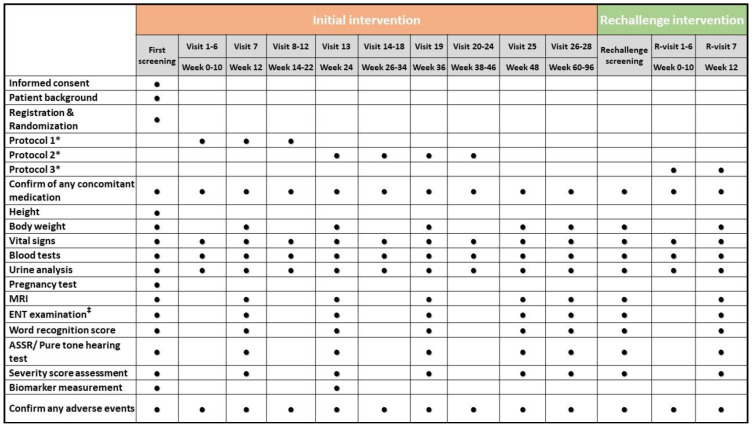
An outline of the study assessments and procedures scheduled. Each circle represents a task or test performed every two weeks. * Description of these protocols can be seen in Figure 2 and Figure 3. ^‡^ ENT examination before the pure-tone test. ASSR, auditory steady-state response; ENT, ear, nose, and throat; MRI, magnetic resonance imaging.

An outline of the study assessments and procedures in the trial is shown in Figure 4.

Patients’ characteristics include gender, date of birth, age, and medical history. The blood tests include a complete blood cell count, renal function test, liver function test, and coagulation profile. The urinalysis includes measurements of protein, glucose, and occult blood. The pregnancy test is performed only in patients of childbearing age. The workup for biomarkers includes free VEGF, plasma hepatocyte growth factor, VEGF-D, and stromal cell-derived factor 1–α.

### 3.8. Statistical Considerations

#### 3.8.1. Sample Size

Based on previous reports, the proportion of improved hearing, assessed by WRS in patients with NF2-related VS treated with bevacizumab, was assumed to be around 50% on average [20,21,31,32,36,37,38,39]. On the other hand, the proportion of improvement in a control group was assumed to be 16% [40]. Accordingly, each group’s required sample size was calculated as 28 patients; this size was needed to provide a power of 0.8 at a significant two-sided α level of 0.05 to detect bevacizumab’s superiority by 34% in the patients with NF2 related VS by a chi-squared test. Taking into consideration the possibility of a 10% sample dropout, the total sample size was upgraded to 60 patients.

#### 3.8.2. Analysis Populations

The following populations for analysis are defined. The efficacy endpoint will be analyzed primarily in a full analysis set and secondarily in a per-protocol set. Safety data will be analyzed in a safety analysis set.

- The full analysis set includes all randomly assigned participants except those enrolled in the study before study approval by ethics committees or execution of essential contracts, those who did not give written consent, and those who never received the treatment protocol;
- The per-protocol set includes all participants in the full analysis set except for those who do not meet the inclusion criteria; those who meet the exclusion criteria; those who have serious violations of the study protocol, such as noncompliance with dosage and administration of study treatment and concomitant medications; those who take prohibited drugs; and those who do not take at least 80% of study treatment;
- The safety analysis set includes all participants who received at least one dose of study treatment.

#### 3.8.3. Statistical Analysis

- The full analysis set population will be used in the analysis of demographic data and other baseline characteristics. For discrete variables, the frequency and proportion will be calculated in each group. For the continuous variables, descriptive statistics (mean, standard deviation, 95% confidence interval) are calculated for each group. Variables to be analyzed include gender, age (at the time of providing consent), height, weight, presence of pre-existing illness, and complications;
- In the analysis of primary outcomes (see Section 3.5.1), the proportion of responders in each group will be calculated, and a chi-squared test will be used to compare between groups. Missing values are imputed with the measured values at the final observation (last observation carried forward);
- In analyzing secondary outcomes (see Section 3.5.2), the variables will be evaluated at the 48 week time. For discrete variables, the frequency and proportion will be calculated for each group. For the continuous data, descriptive statistics (mean, standard deviation, and 95% confidence interval) are calculated for each group. An analysis of covariance will be performed with the baseline or 24 week data as covariates. Missing values are imputed with the measured values at the final observation (last observation carried forward). Concerning continuous data from the rechallenge intervention, the means and standard deviations with 95% confidence intervals will be calculated. A paired-samples *t*-test will be used to quantitatively describe the absolute change from rechallenge initiation to 12 weeks later;
- In the analysis of safety evaluation variables (see Section 3.5.2, item 14), each group’s frequency and proportion are calculated for the discrete data. For the continuous data, descriptive statistics (mean, standard deviation, and 95% confidence interval) are calculated for each group. Missing values are not imputed;
- In the subgroup’s analysis, the primary and secondary outcomes will be analyzed in the subgroups according to the age at onset (≥25 years vs. <25 years) and the number of target lesions (unilateral vs. bilateral);
- In the interim analysis, when all patients reach 24 weeks of intervention, an interim analysis will be conducted to measure the proportion of patients with improved hearing (responders), according to the WRS, at week 24 (day 169) in comparison with the baseline readings.

### 3.9. Ethics

This clinical trial is conducted in compliance with the Pharmaceutical and Medical Device Act of Japan and good clinical research practice. The study will be funded by the FY2019 Clinical Research and Clinical Trial Promotion Research Program from the Japan Agency for Medical Research and Development (AMED). Fukushima Medical University and Chugai Pharmaceutical Co. signed an agreement on 1 April 2019 that Chugai would provide bevacizumab free of charge (IS01001).

## 4. Summary

The BeatNF2 trial, to our knowledge, is the first randomized controlled trial designed to assess the efficacy and safety of bevacizumab in patients with NF2 associated VS. This trial began in October 2019 and is now ongoing. We expect our study results to show significant effects of bevacizumab and provide a new treatment strategy for patients with NF2-related VS.
